# Symptom cluster of pain, fatigue, and psychological distress in breast cancer survivors: prevalence and characteristics

**DOI:** 10.1007/s10549-020-05522-8

**Published:** 2020-01-14

**Authors:** Ellen Bjerkeset, Kari Röhrl, Inger Schou-Bredal

**Affiliations:** 1 Regional Advisory Unit for Palliative Care, Department of Oncology, Oslo University Hospital, Postboks 4956 Nydalen, 0424 Oslo, Norway; 2 Department of Oncology, Oslo University Hospital, Oslo, Norway; 3 Institute of Health and Society, University of Oslo, Oslo, Norway

**Keywords:** Breast cancer survivors, Symptom cluster, Pain, Fatigue, Psychological distress

## Abstract

**Purpose:**

Breast cancer survivors may experience pain, fatigue, or psychological distress as a result of the treatment. These symptoms may co-occur and form a cluster. However little is known about symptom clusters (SCs) in long-term breast cancer survivors. This study aimed to identify subgroups of breast cancer survivors with the SC of pain, fatigue, and psychological distress, and to examine sociodemographic and clinical characteristics associated with this SC.

**Methods:**

Data were obtained from a nationwide survey of breast cancer survivors (*N* = 834). Exhaustive enumeration of possible combination of the three binary variables (pain, fatigue, psychological distress) was conducted. They were identified using the recommended threshold for the Hospital Anxiety and Depression Scale, the Fatigue Questionnaire, and a score of one or more on a numeric rating scale for pain. The SC was defined to include all the three variables, all other combinations were defined as no SC. Logistic regression analyses were conducted to examine the association between sociodemographic and clinical variables and the SC.

**Results:**

Of the 834 survivors, 13% had the SC. Younger age (OR 2.3, 95% CI 1.3–4.1, *p* = 0.003), lymphedema (OR 1.9, 95% CI 1.1–3.2, *p* = 0.02), working part-time (OR 2.9, 95% CI 1.6–5.3, *p* < 0.001), or being disabled (OR 4.1, 95% CI 2.2–7.8, *p* < 0.001) were all associated with the SC.

**Conclusion:**

Thirteen percent of the survivors experienced the SC. It appears that premenstrual women are at greater risk, than postmenopausal women. Having this SC might have an impact on the survivors’ ability to work.

## Introduction

Long-term survival after a diagnosis of breast cancer is steadily rising because of early detection and improved treatment modalities [1]. However, several years after cancer treatment has been completed, a significant proportion of breast cancer survivors suffer from multiple symptoms, of which pain, fatigue, and psychological distress are the most prevalent [2–4]. These three symptoms may co-occur and form a cluster. A symptom cluster (SC) is defined as a stable group of two or more concurrent symptoms that are related to one another, but independent of other symptoms or symptom clusters [5]. A SC may have shared underlying causes and outcomes. Most research focuses on single symptoms, even though the occurrence of a SC appears to worsen patient outcomes. When symptoms “cluster,” they will hasten or potentiate one another through physiological, psychological, behavioral, or sociocultural factors [6]. This collective impact of a SC may have a negative synergistic effect and affect the daily life and function of the patient.

One approach in SC research is to identify subgroups of patients with similar symptom experience with a specific SC [5, 7, 8]. Several studies in both curative and palliative care patients with different types of cancer have found that pain, fatigue, and depressive symptoms constitute a consistent cluster that is often combined with sleep disturbances [7–17]. In addition, research has shown that anxiety and depression also occur in clusters [10, 14, 18]. In the literature, the term psychological distress is often used as a generic term for anxiety and/or depressive symptoms [4, 19]. Psychological distress extends along a continuum, ranging from a normal reaction to a difficult situation to problems that can become disabling [20].

The SC of pain, fatigue, and psychological distress may represent a high symptom burden and has a negative impact on the quality of life and functional performance [5, 15, 21, 22]. Limitations in function may also affect employability [21]. Getting patients back to work is one of the first and most important goals after treatment is completed. However, several studies have shown that breast cancer survivors have an increased risk of unemployment [21, 23–25].

Most cluster research on women with breast cancer has been conducted during the time of treatment or the 1st year posttreatment [7–9, 11, 13, 26]. Thus, little is known about SCs in long-term breast cancer survivors. A common definition of cancer survivors is from the moment of diagnosis and through the balance of his or her life, and long-time survivorship ≥ 5 years [1, 27]. Others define it as patients who have lived beyond 2–5 or 10 years from diagnosis, without recurrence [27]. The last definition is the chosen approach. Comprehensive care for breast cancer survivors includes identifying who has, or is at risk for, SCs. However, in order to identify who is at risk, we need to know which factors are associated with the SC of pain, fatigue, and psychological distress. Previous research has suggested a variety of sociodemographic and clinical factors that predict various SCs, but the findings are inconsistent [11–14, 17, 18].

Research on the concept of SCs is evolving, but still lacks evidence [5, 14, 28]. The literature recommends further research, including research on factors that may predict co-occurring symptoms and the examination of various outcomes in homogeneous groups of cancer patients. To bridge this gap, the purpose of this study is to: (1) identify a subgroup of women who experience the pre-specified SC of pain, fatigue, and psychological distress in Norwegian female breast cancer survivors two to six years post-surgery. (2) Examine sociodemographic and clinical characteristics associated with this SC.

## Methods

### Patients and settings

A secondary analysis of data from a cross-sectional study investigating chronic pain in Norwegian breast cancer survivors was conducted [3]. The Cancer Registry of Norway (CRN) identified a random sample of 1650 survivors who had undergone breast cancer surgery two to six years before the onset of the study. Ten hospitals from different regions in Norway were contacted, and each hospital received a list of patients treated at their hospital from the CRN to evaluate whether they met the inclusion criteria. Exclusion criteria were death, metastatic disease, cognitive impairment, serious psychiatric disorder, or other malignant diseases. Of the 1650 survivors, 1364 were eligible for inclusion. To guarantee participant anonymity, an employee of the CRN sent the questionnaires by mail to the eligible women between October 2009 and April 2010. Thirty-two questionnaires were returned because the recipient was unknown at the address used. The questionnaires were answered anonymously, and the overall response rate was 63% (*N* = 834).

### Patient-reported assessments

Information on sociodemographic and clinical data were obtained by self-reported questionnaires, as well as by multidimensional validated questionnaires to investigate the prevalence of pain, fatigue, and psychological distress.

Pain was first mapped with the question, “Do you or did you have pain after breast cancer surgery and/or during treatment?” If the responder answered “yes,” they were asked to complete further questionnaires identifying various pain characteristics. Pain intensity was measured with the Brief Pain Inventory (BPI) method [29]. The single item “average pain intensity over the past week” was used in the present study. For the BPI, a numeric rating scale (NRS) from 0 (no pain) to 10 (worst imaginable pain) was used. A pain score of 1–3 was rated mild, 4–7 moderate, and ≥ 8 severe [3]. At a score of ≥ 4, it is recommended to conduct a detailed survey to identify clinically relevant symptom burden [30]. The BPI has been translated into Norwegian, validated, and applied on cancer patients (including breast cancer) [31]. In line with the main study [3], chronic pain is determined when a participant answers “yes” to the initial question, and reports a NRS pain score ≥ 1 over the past week.

The fatigue questionnaire (FQ) was used to measure the severity of fatigue [32]. The FQ asks about fatigue symptoms within the last month compared to the conditions when the person last felt well. It consists of seven items related to physical symptoms of fatigue and four items related to mental symptoms. In addition, the FQ has an item assessing duration of fatigue symptoms. Each item is scored by a four-point Likert scale (0–3) where total fatigue is the sum score of all 11 items (33 = maximum score). Each response was then dichotomized (0–1 = 0, 2–3 = 1). Chronic fatigue is defined as a total score of ≥ 4 combined with duration of ≥ 6 months [33]. The FQ has been validated in cancer patients (including breast cancer), and recommended as the preferred multidimensional questionnaire [34] The FQ has also been used to investigate fatigue among breast cancer survivors [4].

Psychological distress was assessed by the Hospital Anxiety and Depression Scale (HADS) [35]. The HADS consists of 14 items covering how the responder has felt over the past week. Seven of the items measure anxiety, and seven items measure depression. Each item has four response alternatives (0–3), resulting in sum scores from 0 to 21. A sum score ≥ 8 within either subscale defines a possible case. The HADS is widely used on cancer patients and has also been applied on breast cancer survivors [4, 36]. Recent studies indicate that HADS does not distinguish adequately between the symptoms of anxiety and depression, but that it is well suited to measure the level of psychological distress [19, 36]. To classify responders with high levels of psychological distress, we merged scores ≥ 8 on one or both subscales into one variable, HADS total [4].

### Statistical analyses

The software IBM SPSS statistics version 22 (IBM Corp, Armonk, NY) was used for statistical analysis. In both the HADS and FQ analyses, missing answers were replaced by an individualized mean score when respondents had answered > 50% of the questions. In addition, failure to answer the question on duration of fatigue was given the value 0.

Age was dichotomized into pre- and postmenopausal age and set to ≤ or > 55 years, according to the National action guidelines for diagnosis, treatment, and monitoring of patients with breast cancer [37]. Body mass index (BMI) was calculated, and—BMI ≥ 30 was defined as obesity. Endocrine therapy was categorized as whether patients had received Tamoxifen or an aromatase inhibitor, or had switched from one to the other. Descriptive statistics with frequency analysis were used to identify sociodemographic and clinical variables, and the prevalence of pain, fatigue, and psychological distress. Bivariate correlation analysis with Pearson *r* was used to examine the strength of the correlations between the three symptoms.

**Fig. 1:**
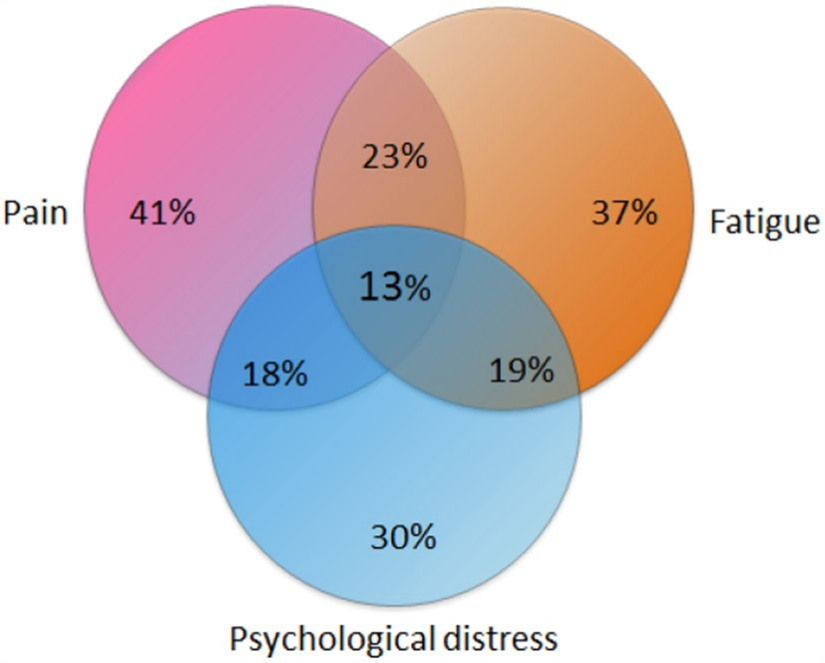
Venn diagram of prevalence and the symptom cluster of pain, fatigue, and psychological distress specified in percent in breast cancer patients 2–6 years post-surgery (*N* = 834)

The HADS and the FQ recommended threshold scores were used to estimate clinically significant psychological distress and fatigue. Women were classified as having pain if they scored ≥ 1 on the NRS and had answered yes on the initial question regarding pain. Exhaustive enumeration at the possible combination of the three binary variables was conducted. Three variables gave a maximum eight. We additionally provide a Venn diagram (Fig. 1) to give another perspective on joint and marginal probabilities/percentage of symptom distributions. The symptom cluster was defined to include all three binary variables: pain, fatigue, and psychological distress.

The Pearson *χ*^2^ test was used for categorical variables, and *t*-tests for continuous variables were conducted to compare survivors with the SC and those without the SC. Logistic regression analyses were conducted to evaluate each of the sociodemographic and clinical variables separately, regarding their association with the SC. Multiple logistic regression was performed to assess the association between the SC and all the variables identified in the univariate analysis using a liberal significance of *p* < 0.25 [38].The Hosmer–Lemeshow goodness-of-fit test was applied to the final model, in which *p* > 0.05 indicated support for the model. The significance level was set at 5%, with a two-sided confidence interval.

## Results

### The SC of pain, fatigue, and psychological distress

Of the 834 breast cancer survivors, a subgroup of 107 (13%) women reported co-occurring chronic pain, chronic fatigue, and high level of psychological distress. Overall, 63% of the women reported one or more of these symptoms 2–6 years post-surgery. The most frequent symptom was pain, 41% reported chronic pain as presented in the main study [3], 37% chronic fatigue, and 30% psychological distress. As shown in Fig. 1, 13% of the survivors had the SC of pain, fatigue, and psychological distress. Of the survivors who reported two co-occurring symptoms 10% had fatigue and pain, 6% had fatigue and distress, and 5% had pain and distress. Few of the survivors reported only one symptom; 13% had pain, but not fatigue or distress; 8% had fatigue, but not pain or distress, and only 6% had distress, but not fatigue or pain. Significant correlations were found for pain and fatigue *r* = 0.49, fatigue and distress *r* = 0.59, and pain and distress *r* = 0.40 (all *p* < 0.001).

Women with the SC scored significantly higher on the NRS measuring average pain intensity over the past week, compared to women without the SC, mean 4.8 (SD 2.2) vs. 1.3 (SD 2.1) *p* < 0.001, respectively.

### Sociodemographic and clinical characteristics

Sociodemographic and clinical characteristics of the women with and without the SC are presented in Table 1. The mean time since surgery for the survivors, who experienced the cluster was 4.1 years (SD 1.5), range 2–6 years. Survivors in the cluster group were significantly younger (*p* < 0.001), had more often received chemotherapy (*p* < 0.001) and/or endocrine therapy (*p* = 0.01), and reported lymphedema (*p* = 0.001). In addition, there was a significant difference between the groups regarding employment status. Significantly more survivors in the cluster group worked part-time or were on sick leave or disabled (*p* < 0.001). No significant differences were found between the groups regarding marital status, education level, BMI, time since surgery, type of surgery, adjuvant radiotherapy, or treatment with Trastuzumab.

**Table 1:** Sociodemographic and clinical characteristics in breast cancer survivors with and without the symptom cluster of pain, fatigue, and psychological distress (*N* = 834)

Characteristic variables	Without SC	With SC	*p*-value^a^
	*n* = 727	*n* = 107	
Age in years: mean (SD)	57.2 (7.8)	54.2 (7.6)	< 0.001
Range	26–74	32–70	
Age group (%)			< 0.001
≤ 55 years^b^	37.1	57.9	
Marital status (%)			0.19
Partnered	71.7	65.4	
Lives alone	28.3	34.6	
Level of education (%)			0.29
≤ 12 years	55.6	61.2	
Employment status (%)			< 0.001
Full time^c^	39.5	22.4	
Part-time	23.4	30.8	
Retired	13.5	5.6	
On sick leave	8.5	14.0	
Disabled	15.2	27.1	
Time since surgery, years: mean (SD)	3.9 (1.5)	4.1 (1.5)	0.55
Surgery (%)			0.78
Breast-conserving surgery	60.3	58.9	
Mastectomy	39.7	41.1	
Axillary dissection	32.9	40.2	0.14
Adjuvant therapy (%)			
Radiotherapy	84.4	86.0	0.67
Chemotherapy	50.8	69.8	< 0.001
Endocrine therapy	55.0	68.2	0.01
Trastuzumab	9.1	10.2	0.72
Lymphedema (%)	17.0	30.2	0.001
BMI: mean (SD)	25.1 (4.1)	25.5 (4.5)	0.33
BMI ≥ 30 (obesity) (%)	11.0	15.0	0.23

*SD* standard deviation, *BMI* body mass index

^a^*p*-value from the Pearson *χ*^*2*^ test for categorical variables, and t-tests for continuous variables

^b^Age ≤ 55 years = premenopausal age

^c^Full time employment is included full time housewife (*n* = 16)

### Univariate analysis

As shown in Table 2, younger age (≤ 55 years) (*p* < 0.001), having lymphedema (*p* = 0.001), and having received combined chemo- and radiotherapy (*p* = 0.04) were significantly associated with the SC. A significant association was also found between the women receiving Tamoxifen and the SC (*p* = 0.03). Obesity was not associated with the SC. Survivors that were disabled had three times the odds ratio (OR) of having the SC, than those who worked full time. Survivors on sick leave had twice the OR of having the SC than those who worked full time.

**Table 2:** Univariate and multiple logistic regression analyses with sociodemographic and clinical variables as independent variables and the symptom cluster of pain, fatigue, psychological distress as dependent variable in breast cancer survivors (*n* = 107)

Variables	Univariate analysis			Multivariate analysis		
	OR	95% CI	*p*-value	OR	95% CI	*p*-value
Age						
≤ 55 years vs. > 55 years^a^	2.34	1.57–3.58	< 0.001	2.31	1.32–4.06	0.003
Marital status						
Partnered vs. alone	1.34	0.87–2.06	0.19	1.53	0.96–2.45	0.08
Level of education						
≤ 12 years vs. > 12 years	0.79	0.51–1.22	0.29			
Employment						
Full time^b^ (ref. category)						
Part-time	2.32	1.33–4.07	0.003	2.91	1.60–5.28	< 0.001
Retired	0.73	0.29–1.84	0.51	1.35	0.50–3.64	0.56
On sick leave	2.91	1.44–5.87	0.003	2.28	1.09–4.77	0.03
Disabled	3.15	1.76–5.65	< 0.001	4.14	2.20–7.80	< 0.001
Time since surgery	1.04	1.04–0.91	0.55			
Surgery						
BCS vs. MAS	1.06	0.70–1.61	0.78			
Axillary dissection	1.37	0.90–2.08	0.14	0.84	0.50–1.41	0.51
Radio- and chemotherapy						
No chemo/no radiation (ref. category)						
Radiation/no chemo	1.30	0.48–3.49	0.61	1.43	0.52–3.98	0.49
Chemo/no radiation	2.98	0.95–9.29	0.06	2.07	0.61–7.07	0.25
Radiation/chemo	2.73	1.06–7.04	0.04	1.80	0.65–5.03	0.26
Endocrine therapy						
No (ref. category)						
Tamoxifen (Tam)	2.13	1.30–3.51	0.03	1.42	0.79–2.53	0.24
Aromatase inhibitor (AI)	1.29	0.54–3.06	0.56	1.38	0.55–3.48	0.49
Switched (Tam + AI)	1.53	0.90–2.60	0.12	1.45	0.80–2.64	0.22
Lymphedema	2.12	1.33–3.35	0.001	1.88	1.09–3.23	0.02
BMI ≥ 30 (obesity)	1.43	0.80–2.56	0.23	1.28	0.68–2.40	0.45

Homer-Lemeshow goodness-of-fit test supports the model (*p* = 0.74)

*OR* odds ratio, *BCS* breast-conserving surgery, *MAS* mastectomy, *BMI* body mass index

^a^ ≤ 55 year = premenopausal age and > 55 year = postmenopausal age

^b^Full time employment is included full time housewife (*n* = 16)

### Multivariate analysis

All the OR`s in the multivariate analysis have a conditional interpretation. In the multivariate logistic regression analyses, none of the treatment variables were associated with the SC (Table 2). Younger age and having lymphedema continued to be significantly associated with the SC. Women who worked part-time had three times the odds of having the SC than those who worked full time (OR 2.91, 95% CI 1.60–5.28). Women who were on sick leave had twice the odds (OR 2.28, 95% CI 1.09–4.77), while women who were disabled had four times the odds (OR 4.14, 95% CI 2.20–7.80) of having the SC than those who worked full time. In the final model, marital status, BMI ≥ 30, the condition of whether or not axillary lymph node dissection was performed were incorporated. The final model was a good model according to the Hosmer–Lemeshow goodness-of-fit test (*p* = 0.74).

## Discussion

### Prevalence

Results from this study shows that 63% of breast cancer survivors report one or more symptoms 2–6 years post-surgery. Chronic pain, chronic fatigue, and high levels of psychological distress, which are defined as a SC, co-occurred in 13% of the women in the present study. Consistent with previous studies on SCs, significant correlations were found between pain, fatigue, and psychological distress [2, 15, 39]. The finding that so many survivors had one or more symptoms contrasts with the “good news” presented by Zucca et al. [39], indicating that only 29% reported cancer-related physical symptoms 5–6 years after cancer treatment. However, in the subgroup of breast cancer survivors, 21% reported two or more co-occurring symptoms, which is in accordance with the findings in the present study. Most breast cancer patients are expected to make good recovery following definitive treatment. However, long-term morbidity associated with breast cancer treatment can be underestimated. This study confirms that a substantial proportion of survivors live with more symptoms than those commonly found in the general population [16, 19, 33, 40]. Thus, we recommend that clinicians should systematically identify symptoms also during follow-up programs and be aware that these symptoms may co-occur and may affect women’s daily life and function.

Symptom clusters including pain, fatigue, and psychological distress have been identified before, during, and after completion of breast cancer treatment [7–9, 11, 13, 17, 26], although there is limited evidence of them in long-term breast cancer survivors. It is challenging to compare our data with results from other studies, because different questionnaires and statistical methods have been used both to identify and classify SCs, and to identify subgroups of breast cancer patients with specific SCs. Several studies have classified subgroups of patients using severity of symptoms scores [7, 8, 13, 26]. Although the group participants in the longitudinal studies vary over time, a stable group scoring high on all three symptoms, defined as a SC before, during, and up to two years after breast cancer treatment, have been identified. In the present study, time elapsed since surgery was not associated with the experience of the SC. Our finding is similar to the findings of Berger et al. [2], who found that time elapsed since diagnosis did not contribute to the symptoms experienced (including fatigue, pain, and distress) in breast cancer survivors, who had completed their treatment up to over 15 years ago [2].

### Sociodemographic characteristics

Previous research on SCs or co-occurring symptoms has suggested that living alone and/or having a low level of education was associated with SCs [11, 13, 17, 22], whereas the findings in the present study did not support this assumption. However, we did find that younger women had twice as high risk of the SC, which is consistent with previous findings [7, 8, 11, 18, 22]. Thus, it is evident that special attention should be given to this age group. Why premenopausal women are at a higher risk for the SC is still unclear. Miaskowski et al. [41] discussed that it could be because younger patients often receive more aggressive treatment. However, we did not find that a combined chemo- and radiotherapy treatment was associated with the SC. It may be biological and age-related changes that moderate the appearance of symptoms, or it may be a “response shift” in older women because of their wider experience and who consider their symptoms to be related to normal aging [41].

Returning to work is one of the first and most important goals after completing cancer treatment for those who were working prior to the diagnosis. Work represents normality and is associated with a higher quality of life [28]. In the present study, there were significantly more survivors with the SC who worked part-time or who were on sick leave or disabled than those without the SC. Cancer-related symptoms such as pain, fatigue, and psychological distress have been associated with unemployment in several studies, but all these studies have investigated one symptom at a time [23–25]. To our current knowledge, the present study is the first to show that the SC of pain, fatigue, and psychological distress after breast cancer treatment is associated with reduced ability to work for many years after the diagnosis. Survivors who were disabled or worked part-time were four and three times more likely to have the SC than women who worked full time. However, due to the study’s design, we cannot conclude that the presence of the SC caused reduced ability to work, only that there is an association between the SC and employment status. There may be several reasons that explain why women were working part-time. Changed priorities after cancer diagnosis can also be a contributing factor. Nevertheless, part-time work has an economic impact because one does not participate fully in the workforce or earn full salary and benefits.

### Clinical characteristics

Except for lymphedema, no other clinical variables were found to be associated with the SC in the present study. Lymphedema is a known and feared late effect of breast cancer treatment. A literature review confirmed that lymphedema affects women’s lives negatively both physically and mentally and includes pain, fatigue, and psychological distress [42]. Our data were collected from 2009 to 2010, and according to the results based on the American College of Surgeons Oncology Group Z0011 trial [43], fewer women now need axillary dissection, which can reduce the risk for lymphedema in a fraction of the patients.

Consistent with previous studies, neither surgery or adjuvant therapy were associated with co-occurring symptoms included in the SC [2, 9, 13, 22]. One reason might be that individual variables that affect symptom experience have a greater impact. Later research indicates that the symptoms of pain, fatigue, and psychological distress may share common biological causes including genetic, immunological, and/or hormonal factors [11, 12, 44]. Future studies should investigate the sociodemographic status and include clinical variables that are disease specific to better understand the effects of SCs.

### Strengths and limitations

The strengths of the present study are that it was based on a large random sample drawn from ten hospitals located in different regions of Norway, thus reflecting the Norwegian breast cancer population. All women were treated according to the same standardized national guidelines [37]. Sociodemographic and clinical variables that in previous studies had shown association with one or more of the symptoms in the SC were included in the present study. Lymphedema and work status had been underutilized variables in previous studies, but were included in the present study. Another strength of our study was the use of validated self-reported multidimensional questionnaires with clinically relevant “cutoff” scores to measure symptoms. In addition, requirements for the duration to define both chronic pain and chronic fatigue were set. Furthermore, this study had taken into account recent research suggesting that HADS is not an accurate enough tool to distinguish between anxiety and depression, but that it is well suited to measure psychological distress [19, 36]. Consistent with previous research on breast cancer survivors, a threshold of ≥ 8 on one or both subscales of the HADS was combined to classify subjects with high levels of psychological distress [4].

The main limitation of this study is the cross-sectional design, which provides only one estimate of the SC of pain, fatigue, and psychological distress two to six years post-surgery. This design did not allow us to draw conclusions regarding causality, it only enabled the description of factors associated with the SC. Other limitations are the lack of data regarding employment status and the prevalence of SC prior to surgery. Several studies have found that comorbidity was associated with a higher symptom burden, but comorbidity was not included in the present study, neither concurrent treatment for symptoms. Insomnia, which has been found to coexist with SCs, was not included in the present study. It might be considered a limitation that information regarding surgical and medical data were obtained by self-report. However, epidemiologic studies have shown that individuals report such data accurately when life-threatening conditions are involved [45]. With a response rate of 63%, it may be a possibility of selection bias.

Our data were collected from women who received treatment for breast cancer during the first decade of 2000, and today’s adjuvant therapy regiments have changed. This might be considered a limitation to the generalization of the findings to today’s breast cancer survivors. However, the present adjuvant treatment regimen still has risks of side effects that might cause late effects in survivors. Furthermore, in agreement with previous research, adjuvant therapy was not associated with the SC in the present study.

## Conclusion

This study found that a substantial proportion of breast cancer survivors experience one or more symptoms of chronic pain, chronic fatigue, and high level of psychological distress 2–6 years post-surgery. Among 13% of the women, these symptoms co-occurred, forming a SC. It appears that premenopausal breast cancer survivors and/or survivors diagnosed with lymphedema have a greater risk for developing a cluster than postmenopausal survivors or survivors without lymphedema. Having the SC might have an impact on breast cancer survivors’ ability to work. The results of the present study support the need for routine screening for multiple symptoms during follow-up care. Improving the identification of those at risk may help guide symptom management strategies and rehabilitation programs tailored to this subgroup of breast cancer survivors.
